# Evolutionary appearance of von Economo’s neurons in the mammalian cerebral cortex

**DOI:** 10.3389/fnhum.2014.00104

**Published:** 2014-03-14

**Authors:** Franco Cauda, Giuliano Carlo Geminiani, Alessandro Vercelli

**Affiliations:** ^1^CCS-fMRI Koelliker Hospital and Department of Psychology, University of TurinTurin, Italy; ^2^Neuroscience Institute Cavalieri Ottolenghi, Department of Neuroscience, University of TurinTurin, Italy

**Keywords:** insula, cingulate cortex, salience network, self-awareness, prediction, development

## Abstract

von Economo’s neurons (VENs) are large, spindle-shaped projection neurons in layer V of the frontoinsular (FI) cortex, and the anterior cingulate cortex. During human ontogenesis, the VENs can first be differentiated at late stages of gestation, and increase in number during the first eight postnatal months. VENs have been identified in humans, chimpanzee, bonobos, gorillas, orangutan and, more recently, in the macaque. Their distribution in great apes seems to correlate with human-like social cognitive abilities and self-awareness. VENs are also found in whales, in a number of different cetaceans, and in the elephant. This phylogenetic distribution may suggest a correlation among the VENs, brain size and the “social brain.” VENs may be involved in the pathogenesis of specific neurological and psychiatric diseases, such as autism, callosal agenesis and schizophrenia. VENs are selectively affected in a behavioral variant of frontotemporal dementia in which empathy, social awareness and self-control are seriously compromised, thus associating VENs with the social brain. However, the presence of VENs has also been related to special functions such as mirror self-recognition. Areas containing VENs have been related to motor awareness or sense-of-knowing, discrimination between self and other, and between self and the external environment. Along this line, VENs have been related to the “global Workspace” architecture: in accordance the VENs have been correlated to emotional and interoceptive signals by providing fast connections (large axons = fast communication) between salience-related insular and cingulate and other widely separated brain areas. Nevertheless, the lack of a characterization of their physiology and anatomical connectivity allowed only to infer their functional role based on their location and on the functional magnetic resonance imaging data. The recent finding of VENs in the anterior insula of the macaque opens the way to new insights and experimental investigations.

## THE ANATOMY OF von ECONOMO’S NEURONS: AREAL AND LAMINAR DISTRIBUTION, MORPHOLOGY, CYTOCHEMICAL CHARACTERIZATION, AND CONNECTIVITY

Large spindle-shaped neurons have been described in layer V of cingulate cortex by Betz (1881) and of frontal cortex by Hammarberg (1895), and later confirmed by Ramón y Cajal (1901-1902, 1904) who first put in evidence their specific belonging to the cingulate and insular cortex. Such cells were occasionally reported in cingular and insular cortex by several authors in the first half of the 20th century, as reviewed by Butti et al. (2013).

**FIGURE 1:**
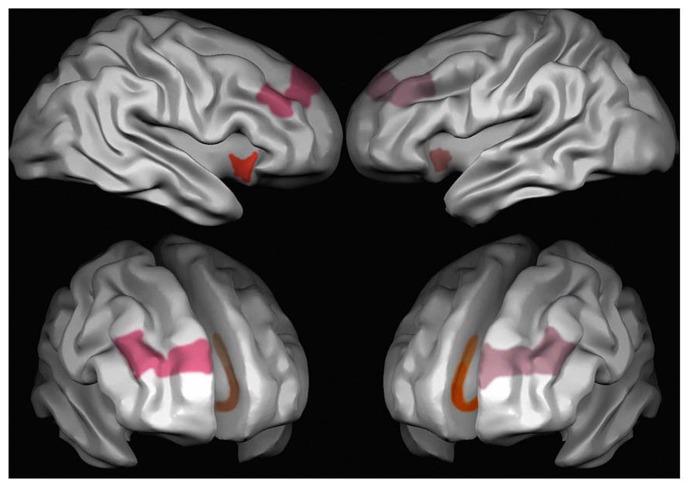
**Areas characterized by the presence of VENs in the human brain.** Red, the anterior insula; pink, area 9 frontal cortex and brown, area 24 anterior cingulate cortex. Different intensities in the colors stand for different densities of VENs, which are more frequent on the right than on the left side in area 9 and in the anterior insula, and in cingulate area 24a > 24b > 24c.

Nevertheless, it was only von Economo and Koskinas (1925), von Economo (1926, 1927) who described in detail their morphology and distribution through the human cortex. For this reason spindle-shaped neurons were named von Economo’s (VENs) thereafter (Allman et al., 2005). VENs were described by von Economo and Koskinas (1925) as large stick-, rod-like or spindle- bipolar/corkscrew-shaped neurons located in layer V of the frontoinsular (FI) cortex, and the anterior cingulate cortex (ACC; Brodmann area BA 24; Nimchinsky et al., 1995; Seeley et al., 2012). Later, VENs have been described in other areas of the limbic system, such as the subiculum and the entorhinal cortex (Ngowyang, 1936), and in the superior frontal cortex (area 9, Nimchinsky et al., 1999). The distribution of VENs in the ACC decreases rostrocaudally (Nimchinsky et al., 1995) in the subdomains of area 24 (24b > 24a > 24c; Vogt et al., 1995), and is more abundant in the FI of the right hemisphere (Allman et al., 2010; **Figure 1**).

The morphology and connectivity of VENs have been analyzed more deeply recently. VENs express neurofilament (Nimchinsky et al., 1995), the peptides neuromedin B (NMB), gastrin-releasing peptide (GRP; Allman et al., 2010) and activating-transcription factor 3 (ATF3), interleukin 4 receptor (IL4Ra), and NMB (Stimpson et al., 2011). VENs also express receptors for vasopressin 1a, dopamine D3 and serotonin 2b receptors (Allman et al., 2005). And finally, VENs express high levels of disrupted in schizophrenia SZ-1 (DISC1) (Allman et al., 2010), which is implicated in neuronal migration during development (Tomita et al., 2011), and are typically disrupted in schizophrenia (Allman et al., 2010). Their size is larger than those of layer V pyramids and layer VI fusiform neurons (Nimchinsky et al., 1995, 1999). Compared to layer V pyramidal neurons, VENs have a vertically oriented basal dendrite, a very limited horizontal extension, and display a low number of dendritic spines (Watson et al., 2006). The vertical orientation of VENs and the narrow lateral extent of their dendritic arbors suggests that they may relay the output of cortical minicolumns (Mountcastle, 1997; Watson et al., 2006; Innocenti and Vercelli, 2010). VENs can be retrogradely labeled in fixed tissue by lipophylic dyes inserted in the white matter of the cingulum bundle (Nimchinsky et al., 1995). For this reason, and since VENs are positive for non-phosphorilated neurofilaments similarly to pyramidal neurons but not to markers of cortical interneurons such as parvalbumin, calretinin, and calcitonin (Nimchinsky et al., 1995), they have been considered as projection neurons (Golgi type I neurons).

The ontogenesis of VENs in humans is difficult to be investigated, due to the lack of specific markers. Actually, the only studies (Allman et al., 2002, 2010) report an increase in the number of VENs in late fetal period (35 weeks of gestation) with a postnatal peak at 8 months, with a decrease in some areas to reach the adult number at 4 years. It is unclear whether this is due to a late differentiation or migration of VENs, whereas layer V should be one of the first layers to form (Rakic, 1974; Zilles et al., 1986). It would also be interesting to investigate the pathway of migration of VENs, i.e., either vertical or tangential, and their origin, either from the subventricular zone or from the ganglionic eminence.

VENs express the peptides NMB and GRP, both of which are involved in the regulation of appetite. Together with the aforementioned expression of high levels of ATF3, interleukin-4 receptor a chain (IL4Ra), NMB, and GRP, proteins involved in gastrointestinal regulation and immune function, this findings led some authors (Allman et al., 2010) to infer that these cells originated in a phylogenetically ancient population of neurons in the insular cortex that are involved in the control of appetite, immune modulation and in the interoception of one’s own homeostatic condition (Stimpson et al., 2011).

## FUNCTIONAL CONNECTIVITY OF THE AREAS CONTAINING HIGH DENSITIES OF VENs

**FIGURE 2:**
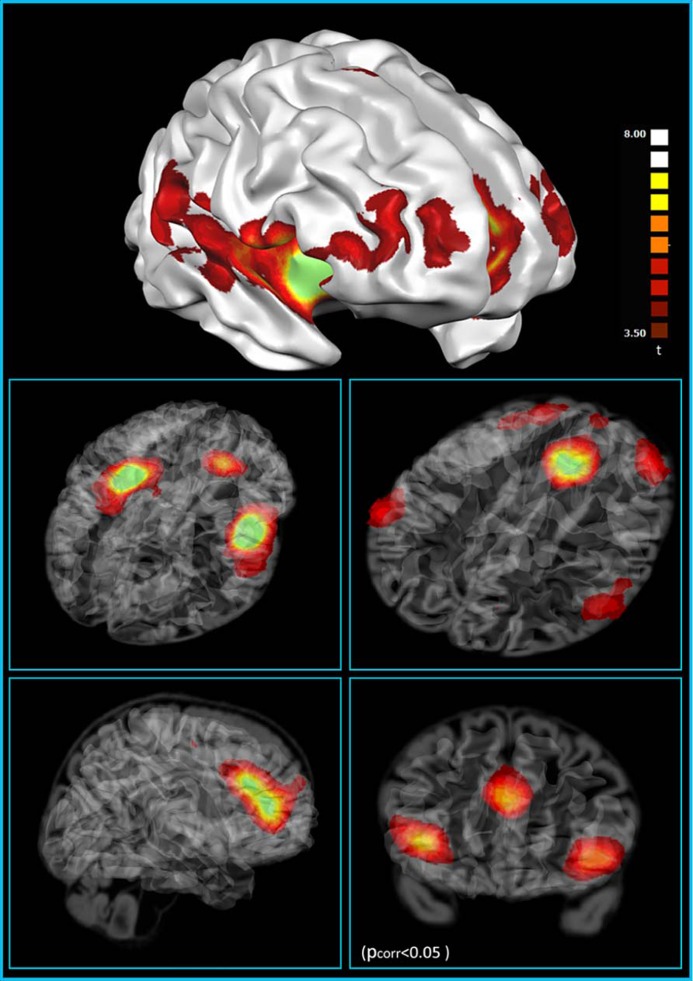
**Resting state functional connectivity of the VEN-containing areas (one sample *t*-test, corrections for multiple comparisons performed at the cluster level using a Monte Carlo simulation; *p* < 0.05), leading to a cluster threshold *k* > 22 voxels in the native resolution; maps are projected on a 3D average brain with use of the Brainvoyager QX surface tool (from Cauda et al., 2012)**.

We have recently investigated the functional connectivity of areas containing VENs. At first, analysis on the functional connectivity of the three ROIs with the highest density of VENs [anterior insula (AI) and ACC] shows that they are part of a frontoparietal network (Vincent et al., 2008; Spreng et al., 2010), including most of the areas of the “saliency detection network” (Seeley et al., 2007a), part of the “control network” (Fox et al., 2006; Seeley et al., 2007a) and part of the network encompassing the posterior insula (Cauda et al., 2011; **Figure 2**). This finding is in line with previous studies that relate the activity of VENs to error monitoring (Dehaene and Cohen, 1994; Seeley et al., 2007a), evaluation of unexpected stimuli, and homeostatic functions (Allman et al., 2010; see Greicius et al., 2003 for a review). Indeed, the AI, one of the areas with a high density of VENs, has been repeatedly found to be active in highly uncertain situations (Seeley et al., 2007a) and saliency evaluation (Menon and Uddin, 2010). VENs are also associated with basic functions of homeostatic regulation (e.g., in the regulation of hunger), and to the homeostasis of social interpersonal relationships. Related to this, it has be noted that VENs are found in species with a highly developed social life (Allman et al., 2010). Our findings on functional connectivity push further the results of the phenotype maps that show that the group of terms that involve salience, theory of mind and social brain are often found together, even though less frequently linked with VENs. The term that is most often used in association with VENs was “social brain” followed by “theory of mind” and “gut.” Moreover, it is interesting, from an evolutionary point of view, to note that the circuits involving areas in which VENs are located comprise ventralmost areas in the frontal and parietal lobes and the insula, which are particularly developed in humans, even compared to great apes (Preuss, 2011).

A recent theory published by Craig (2009, 2010), posit the involvement of the ACC in a plurality of activities such as the evaluation of the emotional aspects of pain, empathy for pain, metabolic stress, hunger, pleasant touch, viewing faces of loved ones or allies, and social rejection (Seeley et al., 2007a). In this view this involvement can be explained if we consider the AI to be a site of convergence for the proprioceptive, interoceptive, emotional, cognitive, homeostatic, and environmental information originating in the posterior insula (Menon and Uddin, 2010). The AI would therefore build a coherent representation of the self in space and time, and the circuit that encompasses the AI would greatly contribute to the awareness of homeostatic changes, either stimulus-driven or stimulus-independent (Craig, 2009, 2010). This and other recent theories relate the activity of the insula to different kinds of awareness (Corbetta and Shulman, 2002; Craig, 2010; Menon and Uddin, 2010), such as motor awareness or sense-of-knowing (Kikyo et al., 2002). Recently Allman et al. (2005), Nelson et al. (2010) implicated VENs in the rapid intuition that relies on an immediate awareness, without the engagement of deliberative processes. These authors therefore specifically relate the VENs, not just to the areas wherein they are frequent observed, but to awareness. Such ability for “insight” is greatly reduced in patients affected by autism (Ben Shalom et al., 2006) and frontotemporal dementia (Day et al., 2013). On the other hand, an hyperconnectivity in the salience network (SN), involving the AI, has been observed in children with autism spectrum disorder (Uddin et al., 2013). Importantly, in the brains of individuals with these disorders, a pathological reduction of VENs has been proposed (Seeley et al., 2006; Santos et al., 2011), perhaps explaining their impaired discrimination between self and other, and between self and the external environment.

Our results (Cauda et al., 2013) also show that functional connectivity between areas with a high density of VENs is not limited to the “saliency-detection” system, but involves other areas of the frontoparietal control network. Recently, Sridharan et al. (2008) demonstrated that the activity of the right AI precedes and causally influences the activity of other areas that belong to saliency and control networks, determining the subsequent state of these two anti-correlated systems. A new theory proposed by Mesmoudi et al. (2013) and based upon some recent functional parcellation papers (Doucet et al., 2011; Cauda et al., 2012; Lee et al., 2012; to cite some), suggest a “dual intertwined rings” architecture of the brain. In this view the resting state brain networks are organized in two families. One with input–output sensorimotor family that includes visual, somatic, and auditory areas and one elaborative and association group that involve default mode, attentional and SNs. Our data on functional connectivity of VEN-rich areas suggest that these areas participate to the second “associative” network.

## INVOLVEMENT OF VENs IN PATHOLOGY

Some data suggest that VENs may be involved in the pathogenesis of specific neurological and psychiatric diseases. VENs are selectively affected (69% reduction in number) in a behavioral variant of frontotemporal dementia in which empathy, social awareness and self-control are seriously compromised (Seeley et al., 2006, 2007a; Seeley, 2008). This reduction in number is specific for this disease, since it does not occur in Alzheimer’s disease (AD; Kim et al., 2012 cerebral cortex). On the contrary, other authors reported a 60% loss of VENs in the ACC also in end-stage AD (Nimchinsky et al., 1995), possibly due to the different stage considered. From a functional point of view, the involvement of VENs could be correlated with apathy when occurring in the ACC.

A reduction in the number of VENs is associated with agenesis of the corpus callosum (CC; Kaufman et al., 2008), while ischemic lesions of the CC do not affect the number of VENs (Allman et al., 2010). The reduction in VEN number correlates with the extent of callosal agenesis, being almost totally absent in the total agenesis of the CC (Kaufman et al., 2008). Further studies would be needed to investigate whether this reduced number of VENs is due to a failure in development or migration, or to increased developmentally regulated apoptosis. The finding that stroke in the CC does not affect VENs supports the idea that their reduced number in CC agenesis is mostly due to developmental defects. Actually, whereas patients with callosal agenesis show, among other symptoms, emotional immaturity, lack of introspection, impaired social competence, general deficits in social judgment and planning, and poor communication of emotions together with diminished self-awareness (Paul et al., 2007), patients in which stroke affects the CC do not. Some of the behavioral deficits observed in callosal agenesis overlap with those reported in autism and schizophrenia.

A decrease in the number of VENs has been implicated in autism as well (Simms et al., 2009); studying a group of autistic patients compared to normal controls, in three out of nine cases an increase in the number of VEN and in six cases a decrease have been reported, and also the dorsocaudal decrease in VEN distribution was altered. This finding is disputed by Santos et al. (2011), who instead reports just an increase in this cell type and by Kennedy et al. (2007) that find no differences in FI VENs between the pathologic and the control group. In any case autism is such a multifarious disorder that might well be compatible with different phenotypes relatively to VENs (Uddin et al., 2013).

Also studies on schizophrenia led to contradictory results. In fact, stereological quantitative studies on the number of VENs in the ACC of patients affected by schizophrenia did not show significant differences between the schizophrenic and the control groups (Brüne et al., 2010). On the other hand, an early onset subgroup displayed a lateralized decrease in VEN number in the right ACC: the protein DISC1, involved in schizophrenia, is preferentially expressed in VENs, and the younger the age of onset of schizophrenia, the lower is the density of VENs in the right ACC (Brüne et al., 2010). This result is in line to the recent demonstration of an aberrant interaction of two large scale brain networks: the executive system, anchored in the dorsolateral prefrontal cortex and the saliency detection system, anchored principally in the right anterior insular cortex in schizophrenic patients (Palaniyappan et al., 2013). Changes in VEN number have been also associated to suicide behavior (Brüne et al., 2011): here VENs are increased in the right ACC, suggesting that an excess of interoception, emotional awareness and self-analysis might be involved in their suicidal behavior.

The finding that VEN’s number is altered in FTD, autism and schizophrenia, developmental and degenerative diseases in which the social brain is affected, further supports a role of VENs in mammals in which the social brain has acquired a specific relevance living in large and socially complex groups (Dunbar, 1998).

## AN EVOLUTIONARY PERSPECTIVE FOR VENs

VENs have been identified in the ACC of the great apes, including bonobos (*Pan paniscus*), common chimpanzees (*Pan troglodytes*), gorillas (*Gorilla gorilla*), and orangutans (*Pongo pygmaeus* and *Pongo abelii*; Nimchinsky et al., 1999). The previous finding of spindle-shaped, VEN-putative neurons in the ring-tailed lemur (*Lemur catta*) and in the chimpanzee (Rose, 1927, 1928) was not confirmed by more recent studies (Nimchinsky et al., 1999). VENs are also found in whales (Hof and Van der Gucht, 2007), and in a number of different cetaceans (with different brain sizes) including the bottlenose dolphin (*Tursiops truncatus*), Risso’s dolphin (*Grampus griseus*), the beluga whale (*Delphinapterus leucas*), and humpback whale (*Megaptera novaeangliae*; Hof and Van der Gucht, 2007; Butti and Hof, 2010); they have also been observed in the brain of the elephant (*Loxodonta africana*, *Elephas maximus*; Hakeem et al., 2009). Their number decreases, as percentage of layer pyramidal neurons in primates, elephants and cetaceans (for a review, see Butti et al., 2013). Of interest is the occurrence of frequent VENs in all cortical areas in the pygmy hippopotamus, a close relative of cetaceans (Butti and Hof, 2010), whereas they are rare in the neocortex of the manatee, a close relative of elephants (Hakeem et al., 2009; Butti and Hof, 2010).

An evolutionary perspective on the involvement of VENs neurons in saliency detection tasks is supported by the finding that the cells are found mostly in animals with a large brain (>300 *g*), but their density is not correlated with relative brain size and encephalization (Allman et al., 2010). In fact, the increase in brain size could causes a conduction delay, i.e., longer time required for the transmission of information, due to the increased distance between connected cell groups. Large VENs, with large diameter axons and high conduction speed, could allow rapid information flow, and would represent an adaptive response to the brain enlargement. Therefore, VENs could provide long-range axons for conveying information as part of a saliency network that may have emerged as a consequence of a larger brain size (Allman et al., 2010). An extension of this hypothesis is that the VENs in FI cortex serve to rapidly relay information to other parts of the brain (Allman et al., 2011). Indeed VENs seem to be especially tailored to convey such information within restricted cortical domains (Buxhoeveden and Casanova, 2002).

**FIGURE 3:**
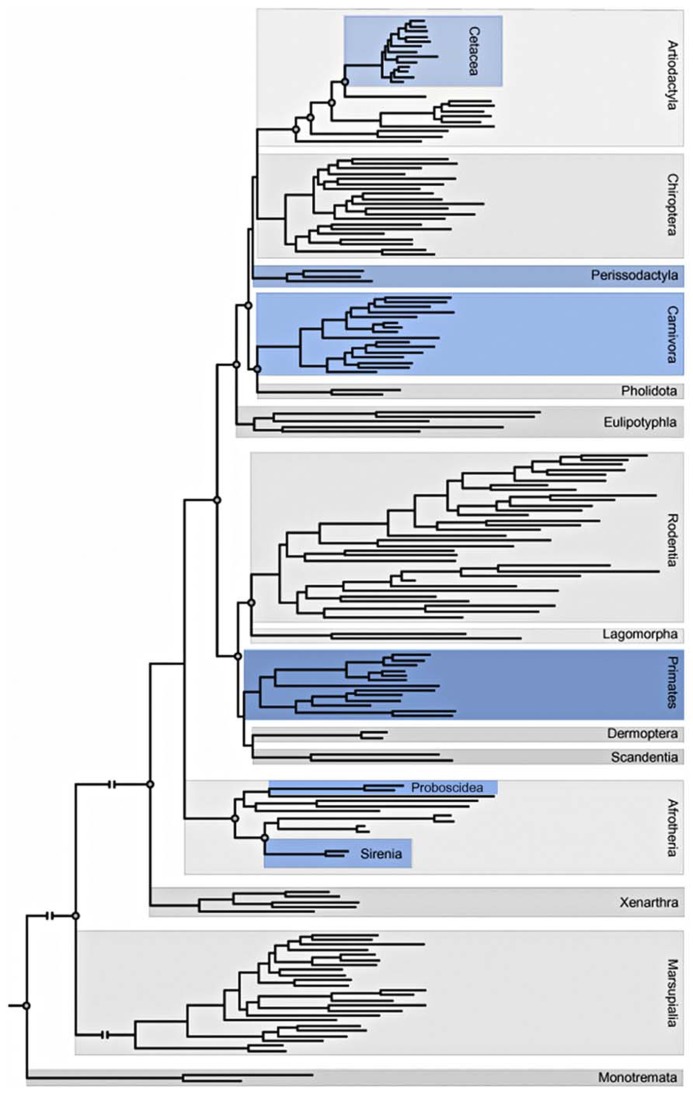
**Adaptation of the phylogeny of placental mammals including Orders and Superorders (Butti et al., 2013; Gatesy et al., 2013).** Blue indicates orders that contain at least one species in which VENs have been described.

This phylogenetic distribution (**Figure 3**) has led to the minimalist hypothesis that the presence of VENs is correlated to brain size; however, others have argued that the presence of VENs is related to special functions such as mirror self-recognition. Additionally, a higher proportion of VENs in human brains are immunoreactive for ATF3, IL4Ra, and NMB compared to the brains of other apes: no other neuron type in layer V of the ACC displays such a significant species difference in the percentage of immunoreactive neurons (Stimpson et al., 2011).

Based on their restricted location and on their specific morphology, VENs would represent the upper motor neurons of the interoceptive system, as well as Betz cells for the motor system and the Meynert cells for the visual cortex.

On the other hand, it has also been proposed that VENs characterize species with common adaptive pressures notwithstanding their divergent evolutionary histories, and which share social, cognitive, and emotional circuits of VEN-containing regions processing fundamental functions for social survival such as strategic communication and the appearance of social hierarchy among members (Hof and Van der Gucht, 2007; Butti and Hof, 2010).

The hypothesis that VENs in humans are implicated in the conscious perception of bodily states and in its integration in conscious decisional processing was initially evoked by Allman et al. (2005). This immediate and complex form of cognition can be also defined as “intuition” or “gut feeling.” The strong labeling of VENs by dopamine D3 and serotonin 2b receptors, involved in signaling the expectation of reward and punishment, respectively (Daw et al., 2002; Sokoloff and Schwartz, 2002), and the strong expression of serotonin 2b receptors in VENs, which can also be found in gastrointestinal cells, was used to support this hypothesis (Baumgarten and Göthert, 1997). The expression in VENs of high levels of bombesin-like peptides, namely NMB and GRP, involved in the peripheral control of digestion and also known to participate in the conscious awareness of bodily states (Allman et al., 2010, 2011; Stimpson et al., 2011), further support this view. The role of the right AI in self-awareness (for review see Craig, 2009) together with the recent findings of Kim et al. (2012) showing that a loss of VENs in the right FI is correlated with symptom severity in bvFTD, indicates that VENs may play a role in interoceptive awareness.

Nevertheless, the increasing evidence for VENs through different species, some of which not closely related to humans, has somewhat challenged to idea of VENs as “the neurons which makes us human” suggesting that they could subserve a more basic role in the networks in which FI and ACC are involved. In fact, recent and very accurate studies reported the occurrence of VENs in the ventral AI of two species of macaque monkeys, rhesus, and cynomolgus (Evrard et al., 2012). This backdates the emergence of VENs in primates from hominids (15 million years ago) to 25 million years ago, at the time of divergence between cercopithecids and hominids (Fabre et al., 2009). The ventral AI of the monkey has been related to both motor and sensory visceral functions (Kaada et al., 1949), which reminds of the visceral activity of the human insula (Craig, 2005). It has been hypothesized that VENs might project to visceral autonomic nuclei, such as the periaqueductal gray and the parabrachial nucleus which are involved in interoception (Craig, 2002; Allman et al., 2005; Seeley, 2008; Butti et al., 2009).

On the other hand, the finding of VENs in the macaque monkeys does not contradict their candidate role in self-awareness and social behavior, as suggested above, even though monkey are probably less aware of the self than hominids (Anderson and Gallup, 2011). Nevertheless, these could be new functions taken up by VENs in addition to their interoceptive, autonomic role during phylogenesis. The occurrence of VENs in macaques will allow to investigate the anatomical connectivity of these neurons by tract tracing *in vivo* (Critchley and Seth, 2012; Evrard et al., 2012). Moreover, this finding should stimulate further studies in other species allowing experimental manipulations (Critchley and Seth, 2012), in parallel with the possibility to investigate their anatomical connectivity in humans with tractography (Jbabdi et al., 2013).

## SUB-NETWORKS IN THE FUNCTIONAL CONNECTIVITY AND LATERALIZATION OF AREAS CONTAINING VENs

**FIGURE 4:**
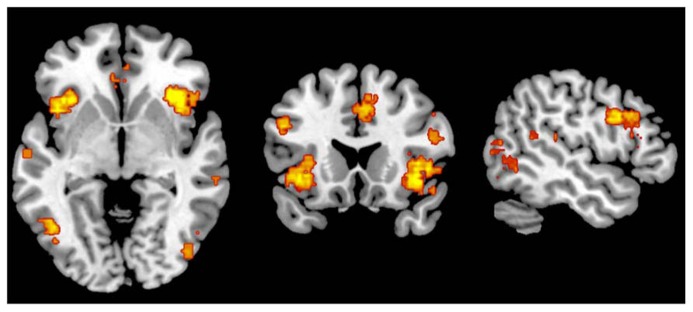
**Metaanalytic representation of the salience network (www.neurosynth.org)**.

**FIGURE 5:**
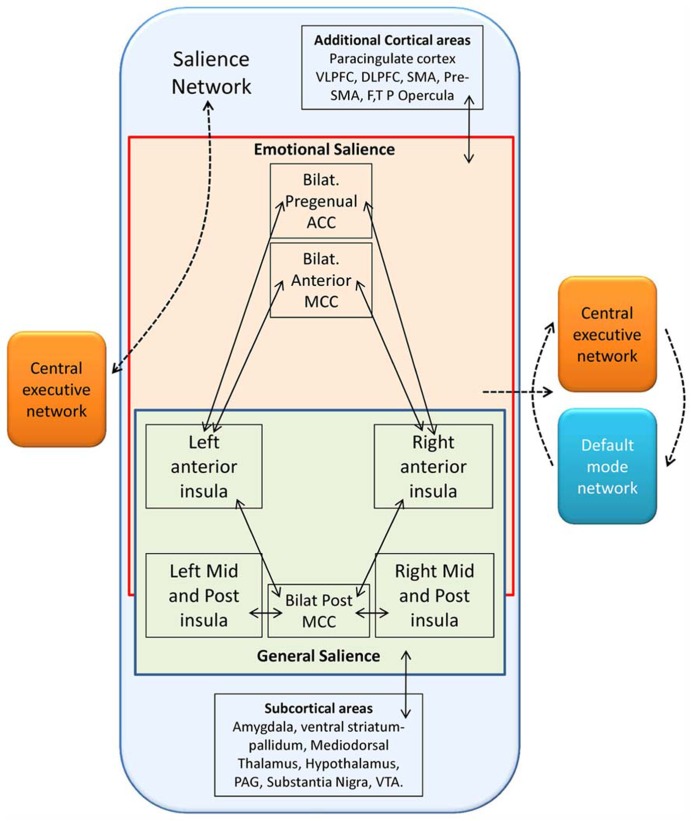
**Schematic representation of functional connectivity in the salience network following Seeley et al. (2007b), Taylor et al. (2009).** The solid lines represent cortical areas found to be functionally connected in fMRI studies (Seeley et al., 2007b; Taylor et al., 2009). The dashed lines represent distinct functional networks and their proposed interactions among these regions. The salience network, characterized by the presence of VENs, can be subdivided in one emotional and one general salience networks both conveying in the anterior insula, whose function as a possible switch node between salience and executive control networks on the left (Seeley et al., 2007a), and as a switch among salience, central executive, and default mode functional networks on the right (Seeley et al., 2007b; Sridharan et al., 2008; Taylor et al., 2009). F, frontal; MCC, midcingulate cortex; P, parietal; SMA, supplementary motor area; T, temporal; VLPFC, ventrolateral prefrontal cortex. Redrawn from Butti et al. (2013).

Recent findings underscore the hierarchical structural organization of cerebral networks, and suggest that the majority of cerebral networks may be further divided into sub-clusters (Bassett et al., 2008; Ferrarini et al., 2009; He et al., 2009; Meunier et al., 2009). This fact is well known to Researchers that perform tract tracing, indeed most cortical areas contain a massive and tight intermingling of neurons projecting to very different brain regions (Zhong and Rockland, 2003; Kennedy et al., 2013; Van Essen and Ugurbil, 2013). In our studies (Cauda et al., 2012), we analyzed the presence of sub-networks in the pattern of functional connectivity of areas with a high density of VEN’s VENs’. By applying fuzzy clustering (Cauda et al., 2013) techniques we divided the network encompassing the AI and the ACC into four sub-networks: the main sub-network was composed of areas of the saliency system (**Figures 4** and **5**), and showed a right lateralization, consistent with the finding of a higher density of VENs in the right insula and cingulate cortex (Allman et al., 2011), and with the report that these cortical areas are thicker in the right hemisphere of normal subjects. Such asymmetry may be explained by Craig’s theory on the asymmetry of the autonomic nervous system (Craig, 2005). In this theory Craig put in evidence that the right hemisphere is more related to sympathetic activation, whereas the left hemisphere is more related to parasympathetic activation; such asymmetry is also consistent with the right lateralization of the saliency detection function that evaluates the potential dangerousness of a stimulus for the survival of the organism (Craig, 2005). The right FI would also play a role in mapping internal arousal and conscious emotional awareness as explained in some recent papers by Craig (2002, 2003, 2009), Critchley (2004). The other three clusters were in part pertaining to the frontoparietal control network, but also of default mode network (Raichle and Snyder, 2007), control network (Menon and Uddin, 2010), altogether these clusters constitute a cognitive ensemble called parieto-temporal-frontal (PTF) ring that is related to attention, language, working memory, motivation and biological regulation and rhythms. The last cluster (Cauda et al., 2011) or auditory-visual, visual-somatic and auditory-somatic (VSA) ring is related to sensorimotor and visual areas (Mesmoudi et al., 2013). These two rings have been recently demonstrated to support a “dual integrative process” were the VSA sensorimotor areas perform fast real-time multimodal integration and PTF areas perform a cognitive multimodal integration (Mesmoudi et al., 2013). In fact, the brain networks constantly communicate with each other and have partially correlated activities (Jafri et al., 2008; Deshpande et al., 2011). Some areas exert a causal influence on the communication between networks, as does AI and central executive network (CEN) on the control and default mode networks (Sridharan et al., 2008; Chen et al., 2013). This causal influence have been recently demonstrated to be modified in patient suffering from schizophrenia (Palaniyappan et al., 2013). It has been suggested that the communication between brain networks may happen through “hubs” (Sporns et al., 2007; Buckner et al., 2009; Zamora-Lopez et al., 2010), areas that are common to two or more networks and that facilitate the transport of information.

Behavioral networks that more frequently activate cortical areas that have VENs are those associated with memory, emotions, attention, interoception, pain and action execution. All of these domains are coherent with the salience processing function and with subsequent activation of effector circuits related to the insula and dorsal cingulate cortex. We found an activation of the AI and of the ACC (Cauda et al., 2012). Indeed, AI and ACC are major components of the system for the flexible control of goal-directed behavior, as recently suggested by Dosenbach et al. (2006). In fact, all studies with functional magnetic resonance imaging (fMRI) paradigm require high attention from the subject. This network for goal-directed behavior is therefore necessarily activated during an activity such as fMRI task that requires sustained attention. It should be mentioned, that although our results cannot be taken as specific for VENs neurons, as the areas under exam present an intermix of different types of neuron, however, in this study we demonstrate that the VEN rich areas have a specific connectivity and probably a hierarchical sub-network structure.

Our data are in agreement with those of other authors, who described an anatomical and functional lateralization in cortical areas containing VENs, already during perinatal development (Allman et al., 2002, 2010).

## PREDICTION, THE INSULA AND VENs

**FIGURE 6:**
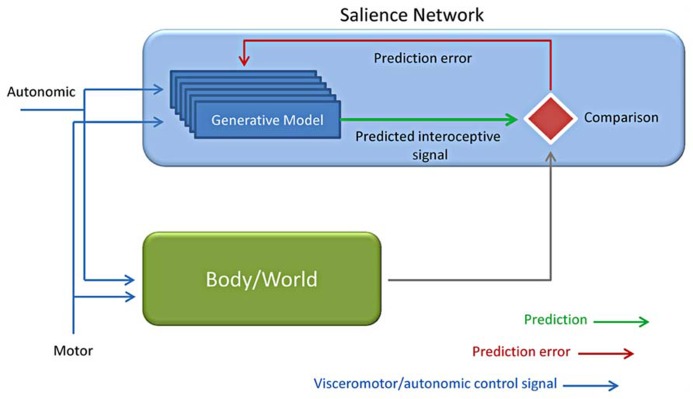
**Predictive coding applied to interoception.** Based on the motor and autonomic control signals, a generative model is evoked together with interoceptive responses from the autonomic control (Body) and the environment (World). The interoceptive predicted (intero pred) responses by the generative model are finally compared to the incoming input by the body /world and used for predicting the consequences of one’s own actions (Adapted from Bubic et al., 2010; Critchley and Seth, 2012; Seth et al., 2012).

The term “prediction,” as well as “preparation, anticipation, prospection, or expectations,” refers to “any type of processing which incorporates or generates not just information about the past and the present, but also future states of the body and of the environment” (Bubic et al., 2010). These terms do not forcedly bear the same meaning, but they can be hardly differentiated. Anyway, all of them underscore the relevance of top-down processing and of how previous knowledge drives and guides present event processing, either of sensory, motor or emotional nature (**Figure 6**).

Interoceptive aspects of emotion led to the theories of the “sentient self” (Craig, 2002, 2009), the “interoceptive awareness” (Critchley et al., 2004) and of the interoceptive predictive model in all of which the insula plays a key role (Critchley and Seth, 2012; Seth et al., 2012; Seth and Critchley, 2013). Some recent works (Ploran et al., 2007; Craig, 2010; Nelson et al., 2010) directly suggest that the insular cortex may be involved in awareness. This hypothesis was initially suggested by Kikyo et al. (2002) who inspected the neural correlates of the “feel of knowing” finding an involvement of the anterior insular cortex. The AI have been proposed to participate in intuition, insight and interoceptive predictive coding (Aziz-Zadeh et al., 2009; Allman et al., 2011). This interoception-related predictive activity is performed by comparing predicted to actual interoceptive signals (Paulus and Stein, 2006; Seth et al., 2012; Seth and Critchley, 2013). Indeed the anterior insular cortex may constitute a possible locus for comparator mechanisms that underly interoceptive predictive coding. This evidence is confirmed by the demonstrated relevance of anterior insular cortex for interoceptive representation and observations reward-related prediction error signals as suggested by findings obtained in different contexts (Singer et al., 2009; Palaniyappan and Liddle, 2012; Seth et al., 2012). Fast connections within the salience detection system and with anterior cingulate and visceromotor systems are preconditions that allow a prompt updating of generative models (Critchley and Seth, 2012; Palaniyappan and Liddle, 2012; Seth et al., 2012). Given the large size of VEN axons these neurons have been hypothesized to provide fast communication between VEN-rich areas and other areas brain (Allman et al., 2011).This hypothesis, although mostly speculative, received an interesting confirmation in a recent study by Chen et al. (2013). In this experiment they demonstrated a directional causal relationship by which a dorsolateral prefrontal node situated within the CEN/SN compound inhibits CEN/SN connectivity with the MPFC portion of the DMN.

Similarly, VENs have been related to the “global Workspace” architecture: according to this hypothesis the VENs are strongly related to emotional and interoceptive signals by providing fast connections between salience-related insular and cingulate and other widely separated brain areas (Dehaene and Changeux, 2011).

## AUTHOR CONTRIBUTIONS

Alessandro Vercelli wrote the manuscript together with Franco Cauda who also prepared the figures. Giuliano Carlo Geminiani supervised the work and revised the manuscript.

## Conflict of Interest Statement

The authors declare that the research was conducted in the absence of any commercial or financial relationships that could be construed as a potential conflict of interest.
